# Supplementary figures and images for: Mutational signature dynamics indicate SARS-CoV-2’s evolutionary capacity is driven by host antiviral molecules
Source: PLoS Comput Biol. 2024 Jan 25;20(1):e1011795. doi: 10.1371/journal.pcbi.1011795 (PMC10868779; doi:10.1371/journal.pcbi.1011795)

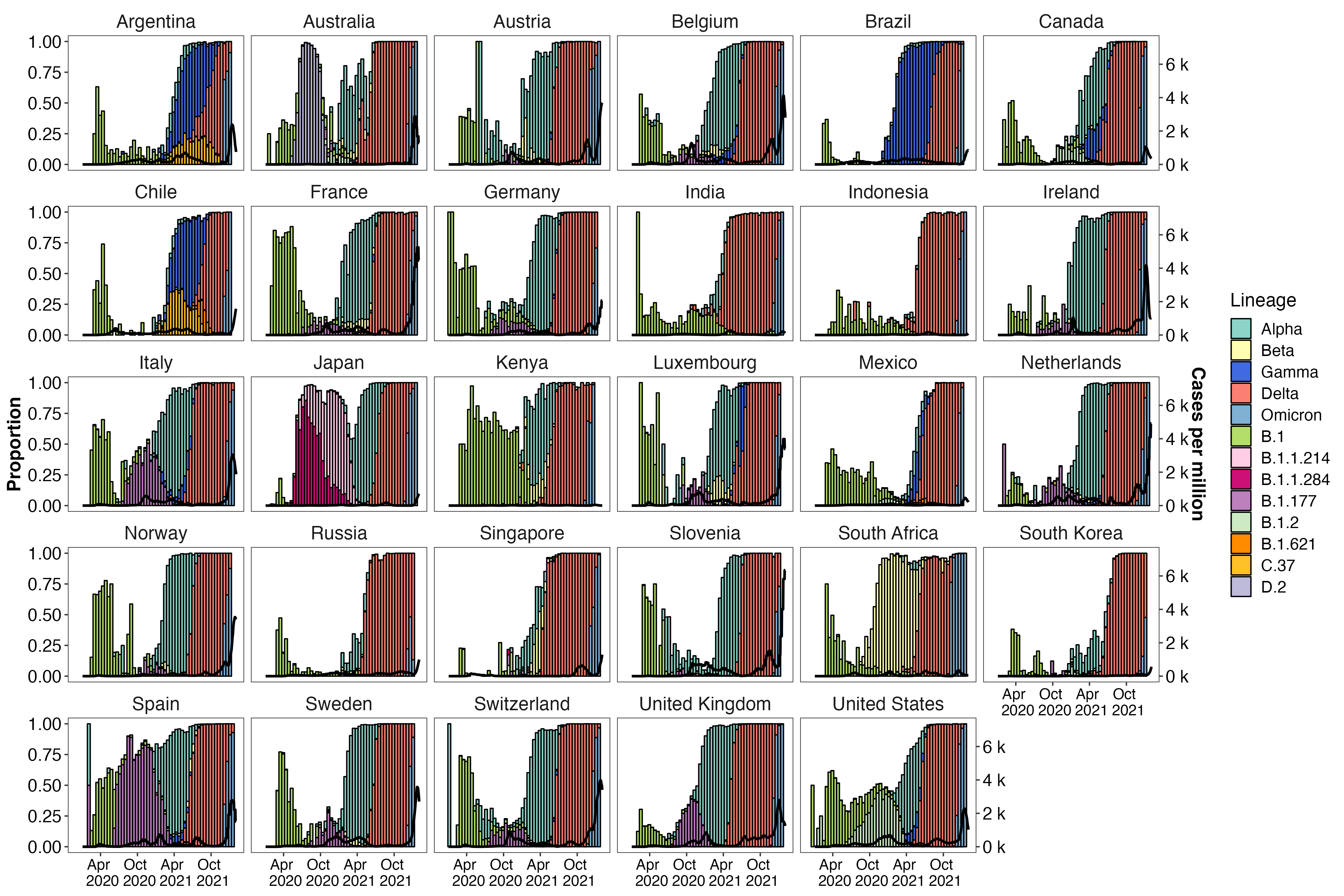

Supplement: S1 Fig — Solid bars show the biweekly proportions of the common lineages. Bars are coloured by lineage and white space shows the proportion of sequences from other lineages. The countries included in this analysis is based on temporal data completeness. (TIF) [file pcbi.1011795.s001.tif]

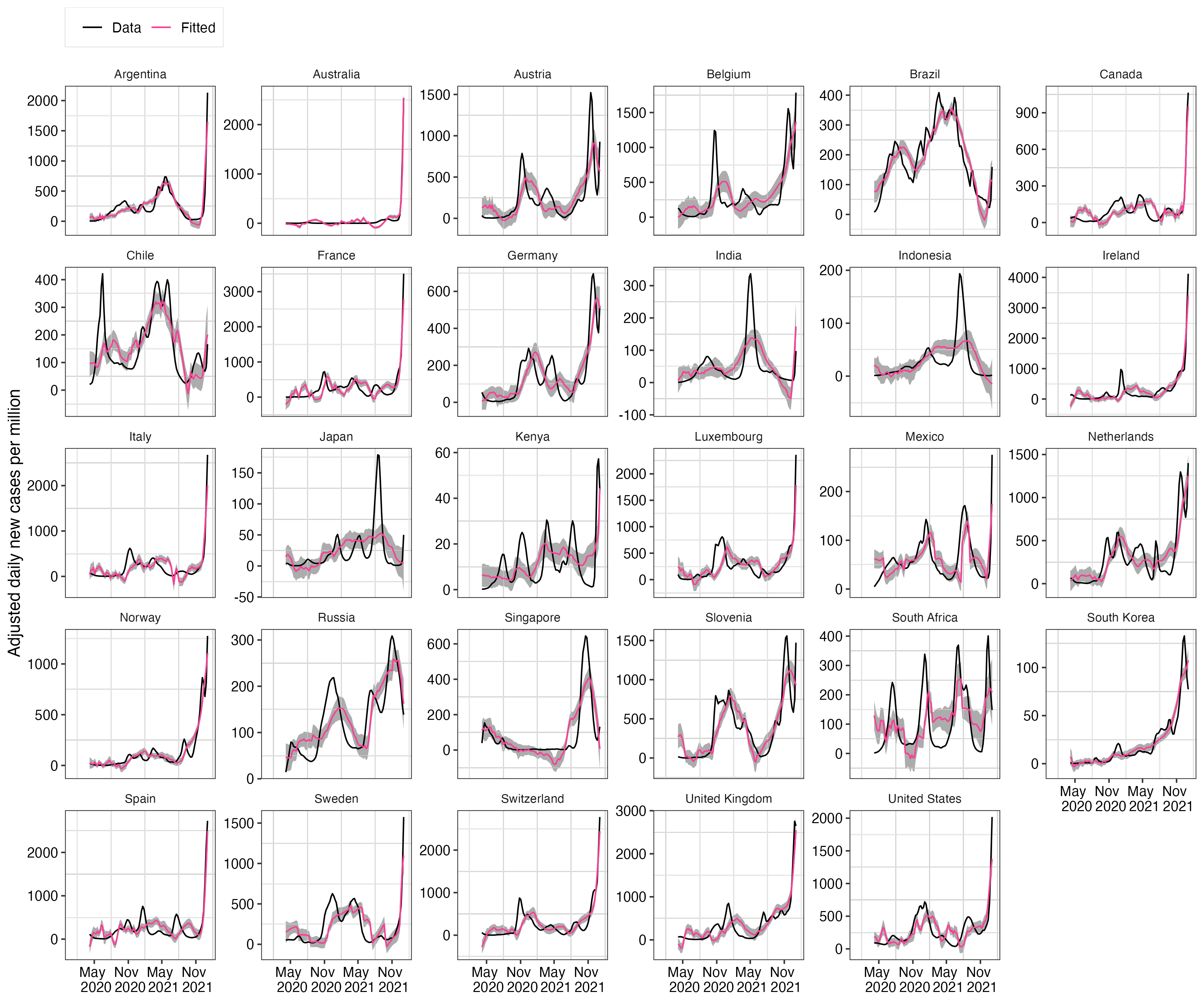

Supplement: S2 Fig — Black solid lines show a 14-day rolling average of adjusted SARS-CoV-2 cases. Pink solid lines show fitted mean response values of infection rates with predictor values as input and grey shaded areas highlight the confidence intervals. The countries included in this analysis is based on temporal data completeness. (TIF) [file pcbi.1011795.s002.tif]

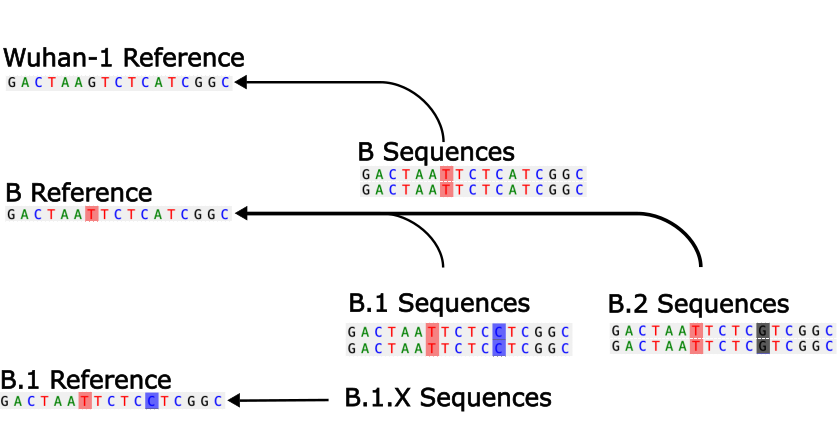

Supplement: S3 Fig — Each Pango lineage has a reference generated for it. Arrows show which sequences use which reference sequence, with the arrow tip indicating the reference. For example, sequences from the B.1 lineage are compared against the reference for the B lineage so that B.1 lineage-defining mutations can be counted. (TIF) [file pcbi.1011795.s003.tif]

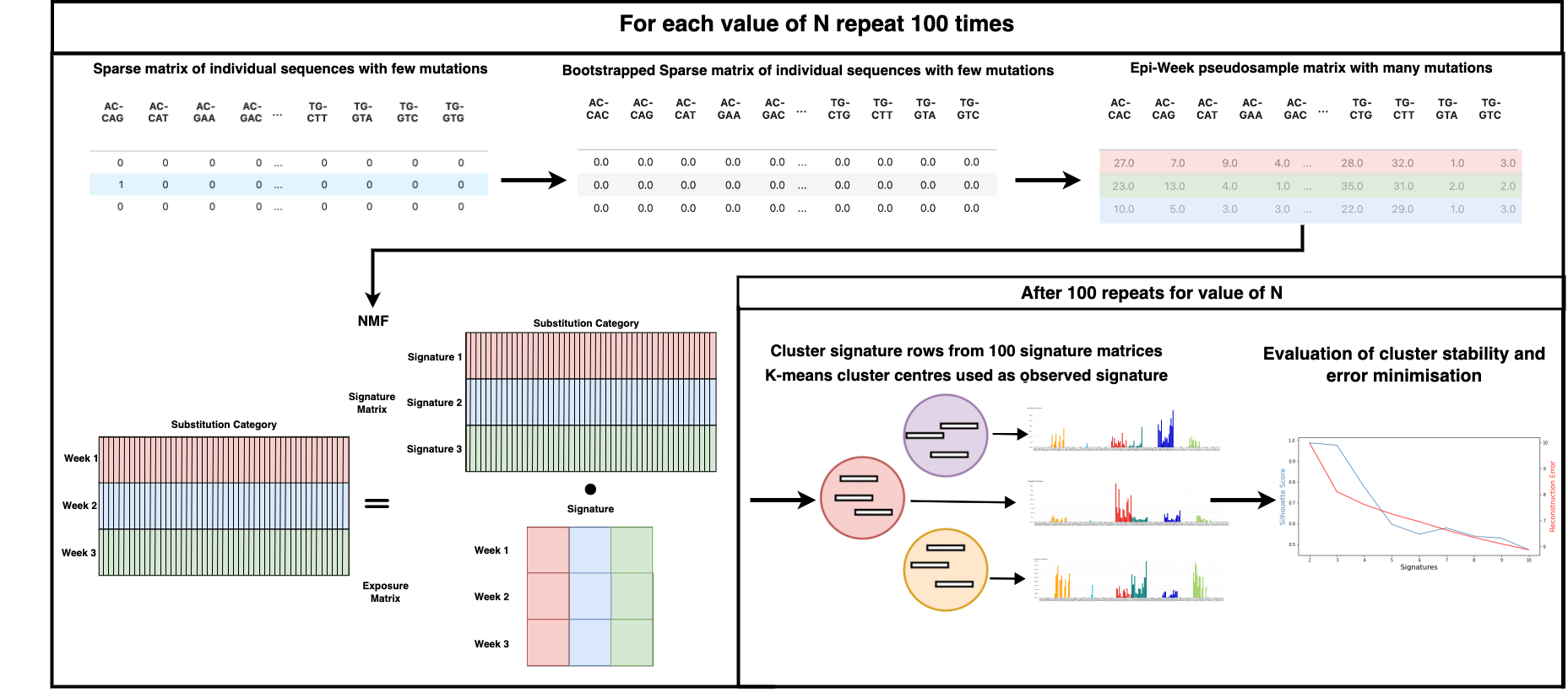

Supplement: S4 Fig — For every value of N signatures, the mutational signatures are extracted 100 times for bootstraped and pseudo-sampled datasets. Once this has been completed, signatures are clustered into N clusters and the stability and density of those clusters are evaluated using the silhouette score. Signatures that have silhouette scores above 0.95 are evaluated as stable signatures. The cluster means become the extracted signatures. The best set of N signatures is selected by picking the value of N that best minimises the reconstruction error and has the best silhouette score (with a minimum of 0.95). A further evaluation is the cosine similarity of the clustered signature means with the signatures extracted by completing NMF on the original pseudo-sampled dataset. Again, signatures must have a cosine similarity of at least 0.95 to be considered. (TIF) [file pcbi.1011795.s004.tif]

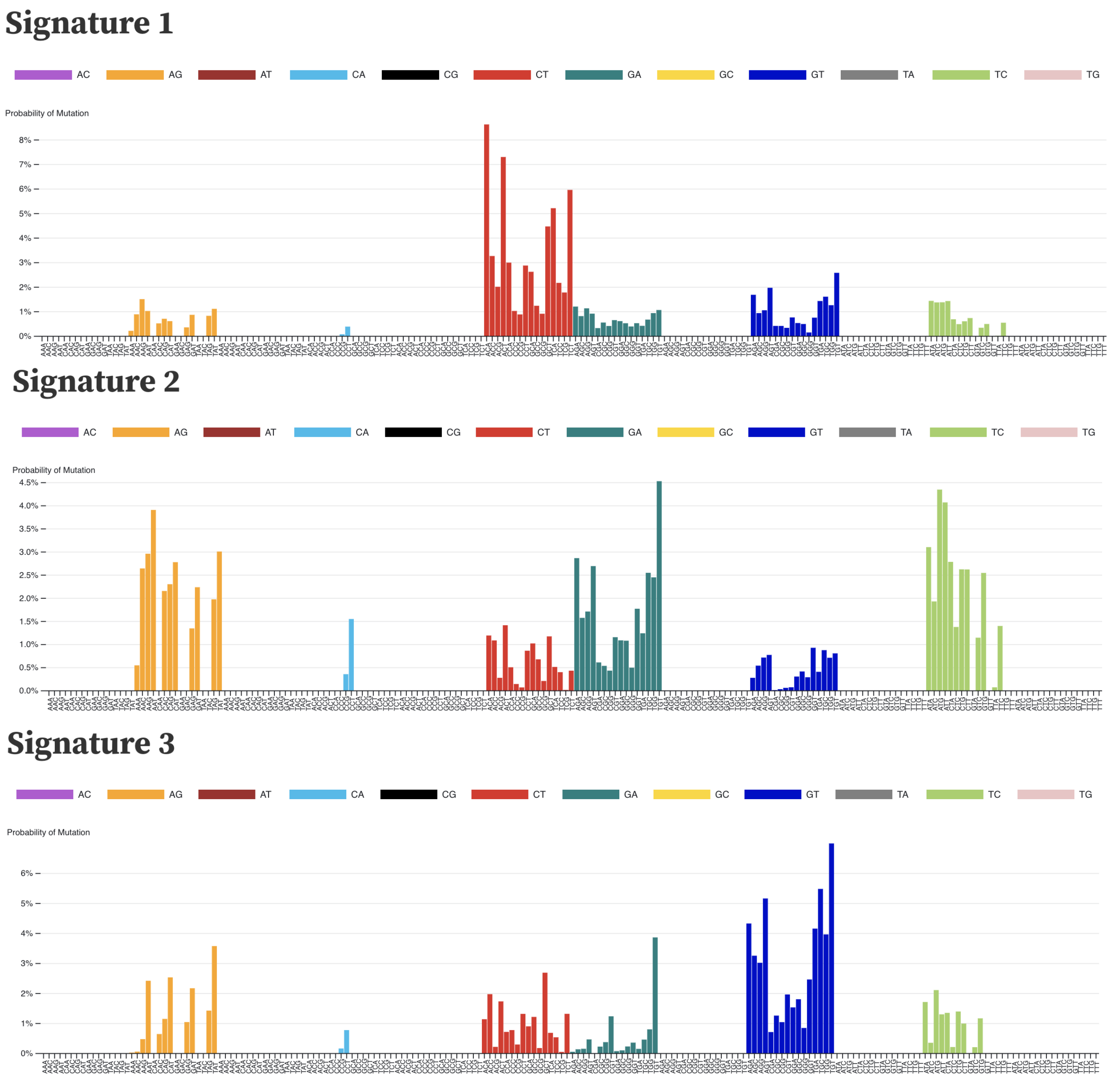

Supplement: S5 Fig — Signatures were extracted using normalised counts calculated by dividing the mutation counts by the count of the tri-nucleotide context of the mutation context (Fig 4). These signatures were then multiplied post-analysis by the tri-nucleotide composition of the reference sequence to produce the non-normalised signatures shown here. (TIF) [file pcbi.1011795.s005.tif]

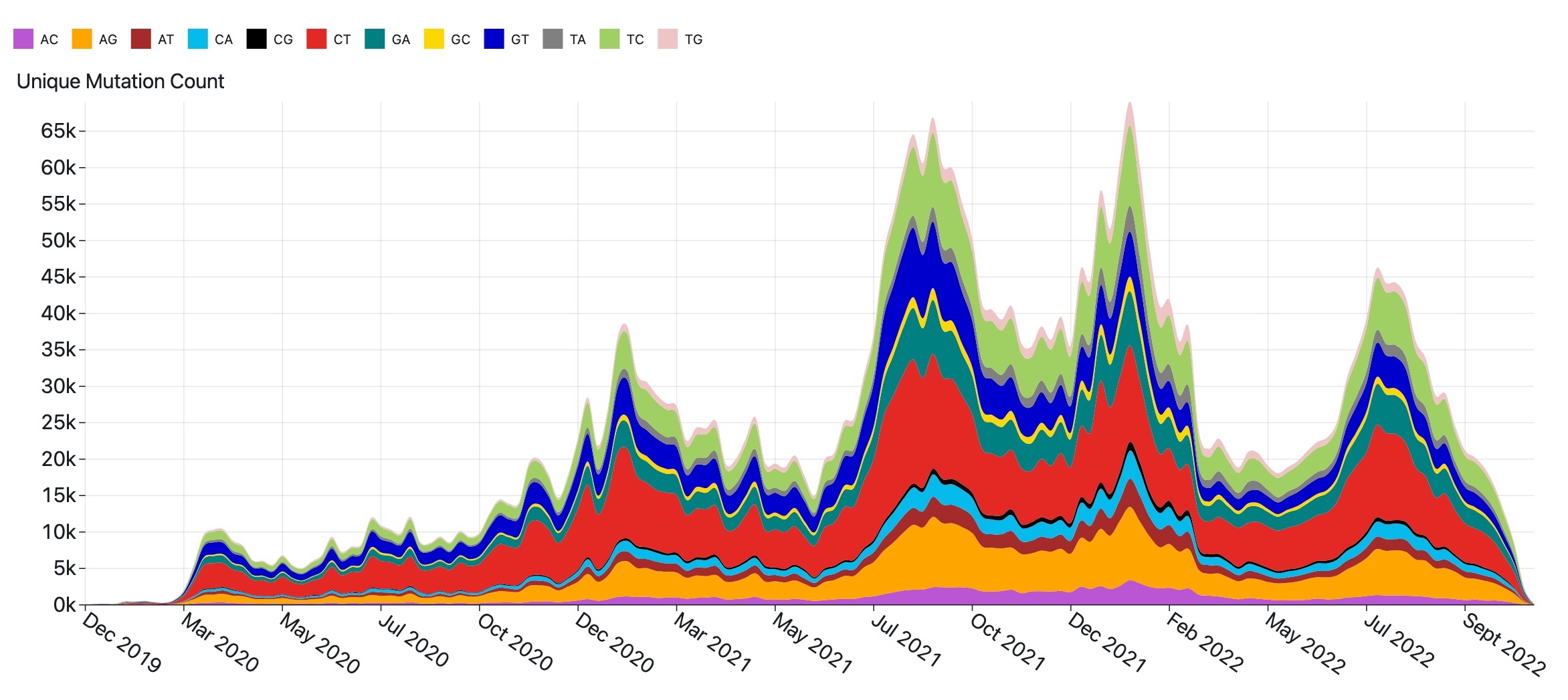

Supplement: S6 Fig — Areas are coloured by substitution category. (TIF) [file pcbi.1011795.s006.tif]

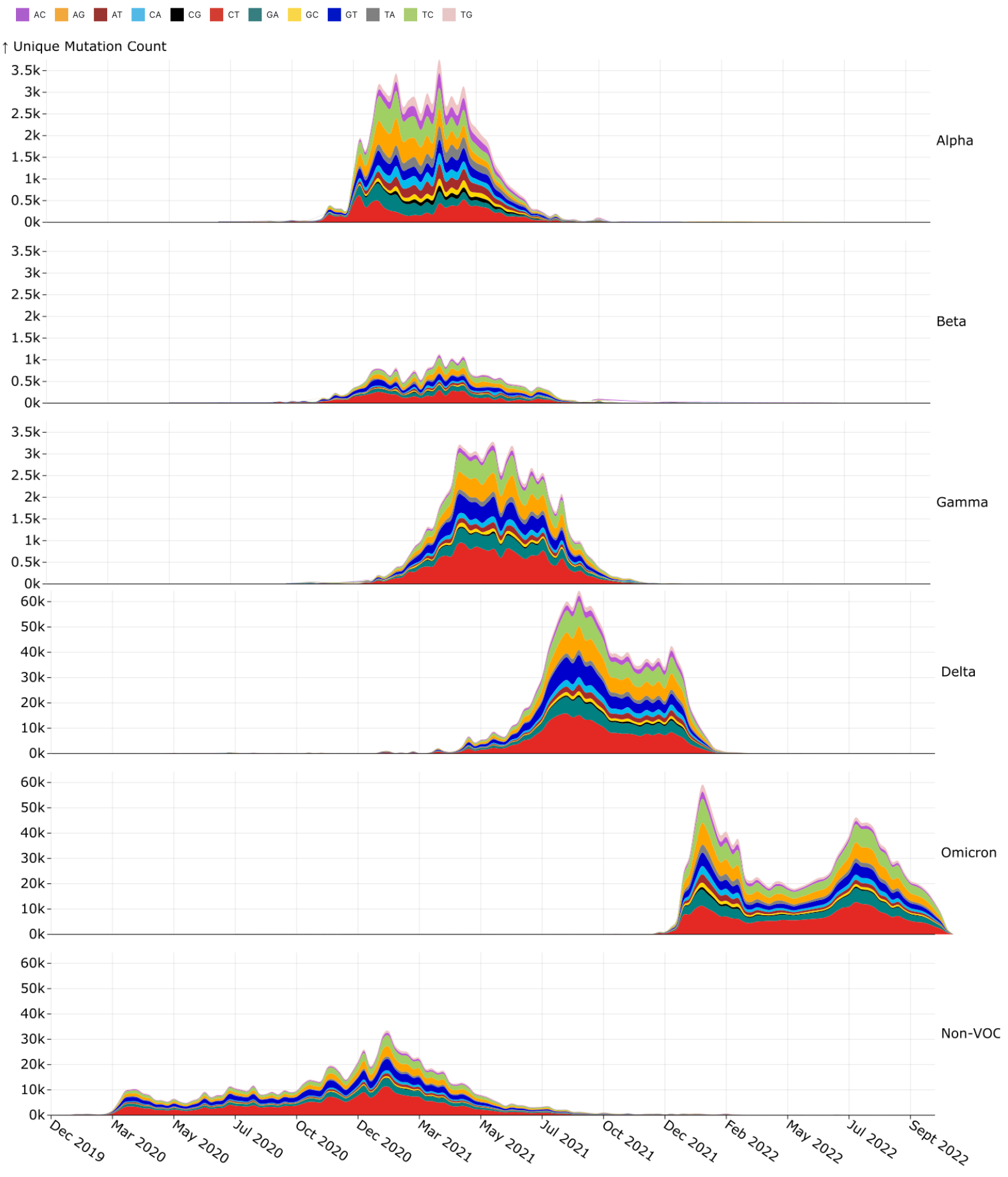

Supplement: S7 Fig — Areas are coloured by substitution category. (TIF) [file pcbi.1011795.s007.tif]

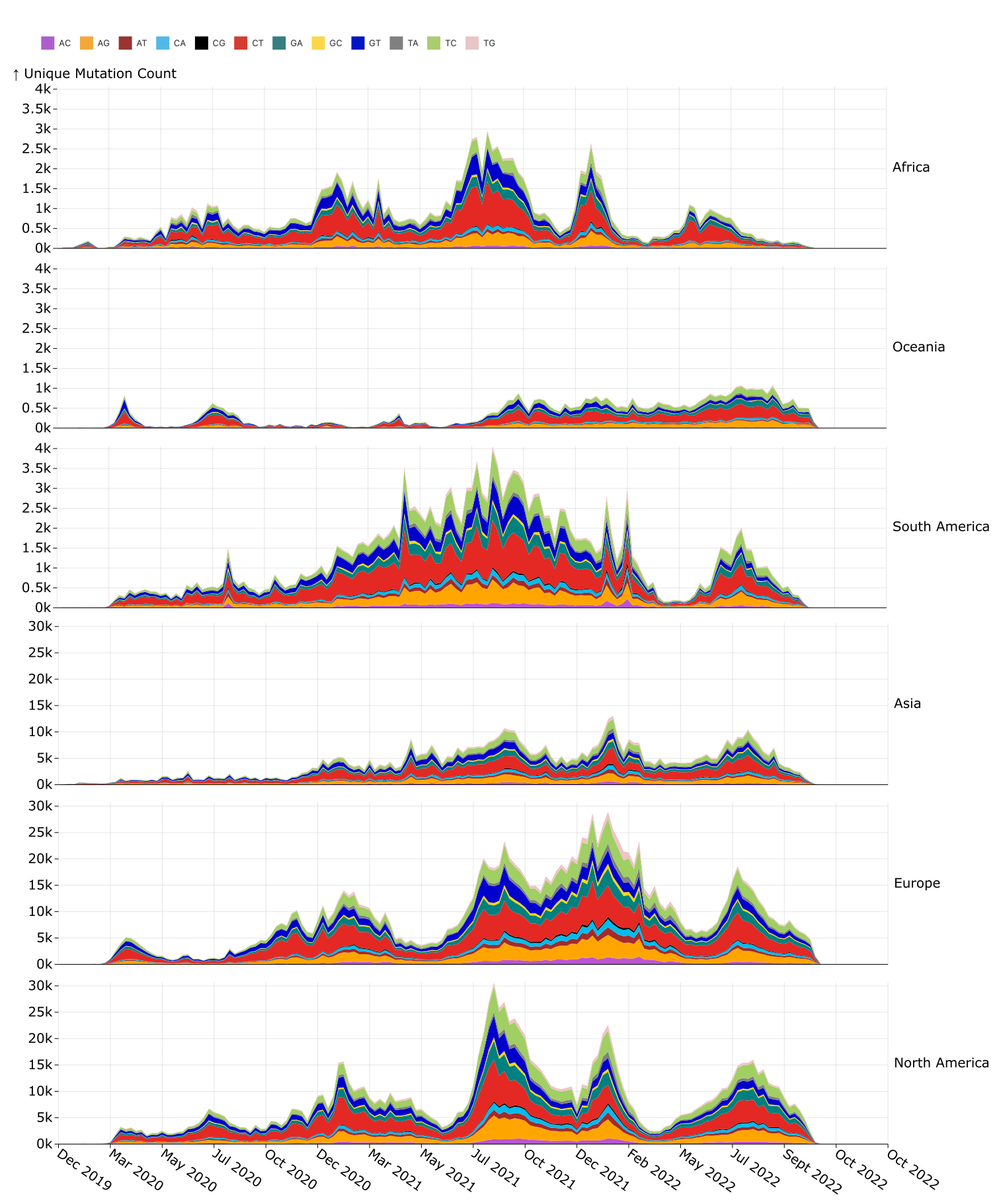

Supplement: S8 Fig — Areas are coloured by substitution category. (TIF) [file pcbi.1011795.s008.tif]

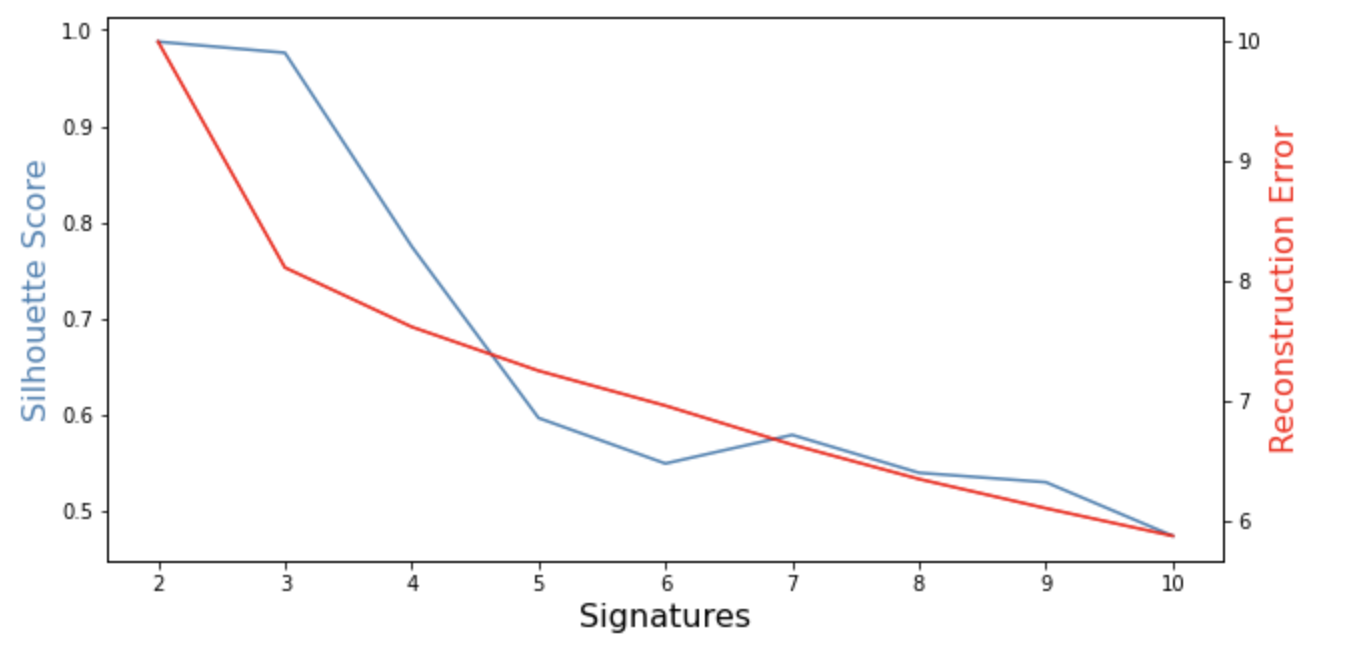

Supplement: S9 Fig — The number of signatures was selected at N = 3 since this produced an “elbow” for the reconstruction error while having a suitable silhouette score greater than 0.95. (TIF) [file pcbi.1011795.s009.tif]
