# Supplementary material for: Mutational signature dynamics indicate SARS-CoV-2’s evolutionary capacity is driven by host antiviral molecules
Source: PLoS Comput Biol. 2024 Jan 25;20(1):e1011795. doi: 10.1371/journal.pcbi.1011795 (PMC10868779; doi:10.1371/journal.pcbi.1011795)
Supplement: Attachment — Submitted filename: Lamb-etal-PLOS_CompBio_SC2- reviewers-response.pdf [file pcbi.1011795.s015.pdf]

Response to reviewers, PCOMPBIOL-D-23-01052

Reviewer 1

Major Comments

Section 2.2 ("covariates of the wave") tries to tackle a very difficult question of what "drives" a SARS-CoV-2 wave. This isn't the key result of the manuscript. This question requires exceptionally careful analysis to answer effectively. In general, the results and its associated methods section are somewhat underdeveloped and do not meet this requirement. This weakness doesn't detract from the rest of the manuscript. That being said, an entire manuscript could be devoted to answering this question and I do not think it is appropriate that the authors change the scope of the manuscript. Instead, I would like to see some small changes to the analysis to increase the robustness of the results and slight dampening of the language in the results and discussion about the impact of this section.

Response: We thank the reviewer for their thoughtful feedback regarding Section 2.2. We recognize the complexity involved in answering the question of what "drives" a SARS-CoV-2 wave. We appreciate the perspective that this section isn't the main focus of our manuscript, but rather an attempt to provide some context and understanding of the factors influencing wave dynamics. In light of this feedback, we:

1. Revise the methods section to be more thorough (e.g., adjusting reported cases by HDI and weighting government stringency by cases) and provide more detail on our approach and make our assumptions more explicit.
2. Undertake additional robustness checks to strengthen our analysis (e.g., reporting R-squared as an indicator of the goodness of fit and using confidence intervals in our predictions).
3. Adjust the language used in the results and discussion sections to be more conservative and highlight the inherent limitations (e.g., limitations of the datasets).

We hope these revisions will address the concerns and preserve the value of the broader work presented in the manuscript
(see additions to pages 4-6).

Both the OWID dataset and the OxCGRT datasets are likely the best datasets to answer the questions posed by the authors but limitations of each dataset should be mentioned in brief in the discussion. For OWID, I would especially call attention to the limitations of "date of report" based time series (see Dong et al. 2022 in Lancet Infectious Diseases for discussion of this).

Response: We acknowledge the constraints inherent to both the OWID and OxCGRT datasets. Discrepancies in parameter definitions, such as varying case classifications (e.g., PCR, antigen, clinical), limited testing capacity for LMICs, and reporting delays, are limitations we've considered. We now discuss this in our manuscript and direct readers to the study by Dong et al. (2022) for a comprehensive understanding of the complexities of these datasets and how they might influence our results (see additions to page 21).

The regression of daily cases to government stringency index needs to be adjusted for the latter metric to be informative. The continent-level 14-day moving average is an appropriate means of dealing with the noisiness of reported case data, where there are frequent gaps in reporting that appear to be zero daily cases. The inappropriate step in this analysis is comparing the continent-level 14-day moving average of new cases to the average OxCGRT index of the component countries within the continent. The daily case rate will naturally be biased toward the countries with the highest case numbers (who contribute the greatest proportion of cases to the average) while the average OxCGRT index will have equal contributions from each country. In a scenario where only a handful of countries have low stringencies indices and these same countries are responsible for an outsized number of cases, any positive effect of government stringency will be dampened (as the OxCGRT index will effectively be artificially inflated). I highly recommend repeating this analysis but weighing a country's contribution to the continent-level OxCGRT based on its contribution to the continent-level 14-day average daily cases within the same time window. From my assumptions of how the analysis scripts have been written, I don't anticipate this being too onerous of a calculation. However, if this is challenging other approaches could be considered. I recommend this approach rather than weighing by population, as the former will still partially control for differences in population (India will be weighed more heavily than Nepal, for instance) while the latter will have the issue of more populous countries not necessarily having infrastructure for widespread community testing.

Response: Thank you for the excellent suggestion to weigh the stringency index by each country's contribution to the number of cases. We agree that this addresses countries with an outsized number of cases to avoid artificial inflation. In response, we have refined our analysis, now weighting each country's influence on the continent-level OxCGRT index according to its proportionate impact on the continent's 14-day rolling average daily case count (Figure 2B), see additions to page 6.

This does not need to be adjusted by the authors, but for the sake of documentation part of the "careful analysis" referenced above would include empirical testing of the assumption "Sequences reported in GISAID were assumed to be representative of the diversity of infections for that continent/country." Performing these analyses is not key to the interpretability of the later results section and for that reason I don't consider it necessary.

Response: We appreciate this insight regarding the representativeness assumption of sequences from GISAID. Although we haven't validated this assumption in this work, we acknowledge its significance and consider it an important direction for subsequent research or analyses.

The authors make a very astute observation on page 18 that the ROS-like signature appears to have context preference: "The G→T signature itself seems to display a context preference of TpG and ApG nucleotides". This observation can (and should) be tested quantitatively. The same approach as in PMID: 34061905 can be used to calculate normalized context preferences. As one will need to compare to a reference sequence, I would approach this by

calculating ratios for each tree-based reference for the pangolin lineages (using the observed substitutions for those references) and then calculating a median ratio from all of the values. This way, you'll be able to robustly determine whether or not any contexts are over- or underrepresented. Dinucleotide context is probably sufficient, but if it is computationally straighforward then trinucleotide context would also be useful and interesting. The reason I view this as key to the manuscript is that the observation of a context preference for ROS would either (a) indicate context and structural preference for ROS, which would be a novel finding or (b) bolster the argument that the G—>T phenotype may be due to another as of yet defined nucleic acid editing mechanism. Either of those findings would be highly significant to the overall story and for that reason deserve robust statistical analysis.

Response: Thank you for the excellent suggestion. We have investigated the mutational signatures using normalised counts for each substitution since our current signatures did not account for context biases from the reference. For this normalisation we divided our initial counts by the number of available triplets in the reference to allow for context-agnostic signatures. With regards to the G->T signature we find that the TpG and TpA context preference do appear to be driven more by their abundance in the genome, rather than any specific targeting (see additions to page 18) and revised figure Suppl. Figure S5:

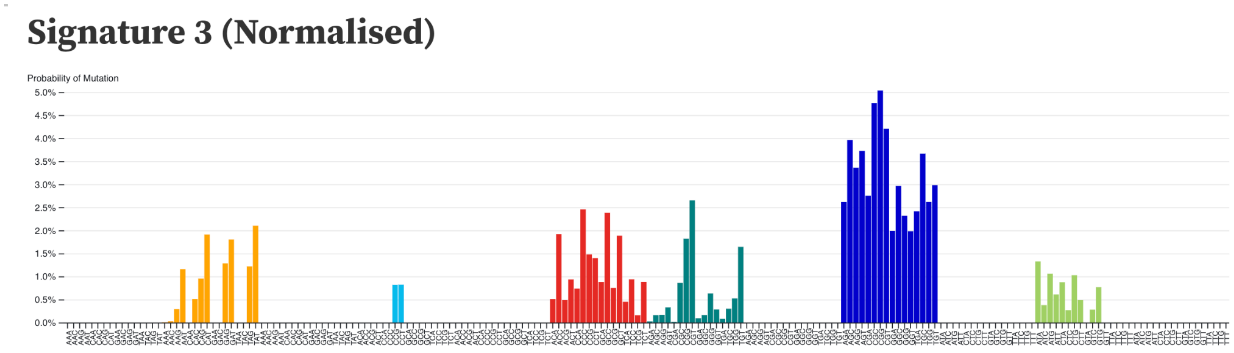

In pages 17 and 18, the discussion of potential molecular mechanisms underlying the (lack of) ROS-like signatures in Omicron is excellent. I am very interested in what hypotheses and explanations the authors are able to generate for the preponderance of the ADAR-like signature in Alpha. The data in figure 5 is particularly striking and I think this result deserves more attention in the discussion that it is currently given.

Response: Thanks for highlighting this, we agree this is an interesting result and have expanded our discussion around it on page 19.

On a smaller (but important) note, additional clarification is needed on page 20 in the methods to ensure reproducibility. Please state on which date the daily case data was downloaded from Our World in Data's GitHub repository.
If prior to February 20, 2023, the OWID data was actually just piped from the Johns Hopkins University COVID-19 dashboard - there are several publications that can be cited about this work (Dong et al 2020 and Dong et al 2022). If after that date, the case data comes via the

World Health Organization. Recognizing the original source is important as the two projects had different approaches to assigning dates to historical cases when historical revisions were made publicly available. These, in turn, impact when the daily cases appear in the case time series. Additionally, the historical case data on OWID is under revision so it is possible the associations reported in the manuscript may change if the analysis were repeated in the future. With a download date, individuals could look at commits to the OWID GitHub repository and use case data from the same date as in this manuscript.

Response: Our data was sourced from the Our World in Data's GitHub repository in September 2022, but we were primarily interested in data until January 2022 for model fitting. Up until 20th February 2023, the OWID data primarily sourced its information from the Johns Hopkins University COVID-19 dashboard, as highlighted in publications by Dong et al., both in 2020 and 2022 (we now cite the two papers). We also acknowledge that OWID's historical case data is undergoing revisions, which could potentially alter associations should the analysis be replicated in the future. With our specified download date, researchers can cross-reference commits to the OWID GitHub repository and use case data corresponding to the same date as in our manuscript (see page 20).

Minor Comments

- The use of the oxford comma throughout the manuscript is inconsistent. Please screen and add or remove, depending on author preferences.

Response: This has been addressed.

- Page 4, paragraph 1: There is an unmatched closed parenthesis (")") in this sentence.

Response: This has been addressed.

- Page 4, paragraph 1: The statement "Third, virus fitness was associated with high infection numbers in all continents except Africa (no significance)." does not match the visualization in figure 2 where virus fitness has a negative association in North America.

Response: Thank you for pointing out the discrepancy between our statement on page 4, paragraph 1, and the visualization in Figure 2. We have now redesigned our model to be more robust and revised the language to accurately reflect the findings.

- Page 5, paragraph 1: I believe "Supplementary Figure A1" is meant to refer to "Figure 1" and "Supplementary Figure A2" is meant to refer to "Figure 2". Please confirm.

Response: This has been addressed.

- Page 8, figure 3 caption: missing a parenthesis after "Simmonds et al"

Response: This has been addressed.

- Page 9: Please use a "substantial" instead of "dominant" to refer to the ADAR signature. I think "dominant" is slightly confusing as the most dominant signature remains APOBEC.

Response: This has been addressed.

- Page 10, figure 5: I want to note that I found this figure very interesting and well-presented. There are a few small items to fix. In panel B, would it be possible to change the country names away from all capitals and remove the underscores for North and South America? In panel D, the y axes dynamically fit the data for all continents except Asia. Can that be adjusted?

Response: This has been addressed.

- Page 10: I don't believe Ruis et al should be cited as "Ruis, Peacock et al"

Response: This has been addressed.

- Page 11, section 2.5: The paragraph is interesting, but it doesn't need to be stated twice to be understood (it's currently duplicated).

Response: This has been addressed.

- Page 17: Please be consistent with writing out "negative sense" or abbreviating as "- sense"
Response: This has been addressed

- Page 17: ROS has already been defined on page 7

Response: This has been addressed.

- Page 17: I would recommend reading and citing PMID: 34061905 when discussing ROS. Ansari and Simmonds only found a ROS-like signature in coronaviruses and not other RNA viruses, which is interesting.

Response: We have included the citation and mentioned it during our G->T discussion.

- Page 19: Be explicit as to whether the statement "Synonymous changes were much more likely to occur in ORF1a/b, which would be expected due to its size as the largest ORF," is referring to counts of synonymous mutations or rates of synonymous to non-synonymous mutations. I could agree with the former, but don't quite believe the latter.

Response: We have changed this to hopefully more explicitly state that this has to do with counts of mutations rather than with rates.

- Page 19: Replace “bu” with “by”

Response: This has been addressed.

- Page 19, paragraph 4: Would the authors be able to provide a bit more detail as to how sequences with “excessive mutations/deletions” were identified and screened out?

Response: The cutoff for filtering out hypermutated sequences was 175 mutations in coding regions or more than 69 different deletions, the cutoffs were manually determined after evaluation of the mutation/deletion distribution and selecting the point where sequence counts were consistently observed in single digits, this resulted in 1,852 sequences being filtered out.

- Page 20, paragraph 4: There is a missing parenthesis at the end of “log(total mutations per week” (in the equation)

Response: This has been addressed.

- Page 32, figure: Please change the y-axis to “Daily new cases”

Response: This has been addressed.

Reviewer 2

Major Comments

The three signatures that the authors have extracted are novel and interesting, as are the dynamics of these signatures through time. However, the assignment of these signatures to drivers based simply on their dominant substitution type is highly problematic. For example, there are a large number of processes that can cause C-to-T mutations (for example see PMID: 30982602). APOBECs are one such process but it is not sufficient to identify C-to-T mutations and jump straight to APOBECs as the cause. Likewise, there are other factors that can cause G-to-T mutations apart from ROS. Signature 2 consists of A-to-G and T-to-C mutations, which are symmetric so the authors assume ADAR; why would this signature not be consistent with a change in polymerase error profile or another mutagenic factor that causes both substitution types? The assignment of signatures to causes is a major issue with the manuscript currently as it drives a lot of the narrative, including the title. But there is not any support provided for these drivers over any other driver of these mutation types. It is reasonable to speculate that APOBECs, ADAR and ROS may contribute to the mutational burden of SARS-CoV-2 but there is not evidence to show that they are major drivers and the conclusions of the manuscript in this

regard are therefore not supported. I'd suggest that the authors refer to the signatures throughout as, for example, 'signature 1' rather than 'APOBEC-like'.

Response: We take the reviewer's point and have taken a number of actions to address these comments:

1. Throughout the results, signature labels were changed from "APOBEC-Like" to "Signature 1" so that an individual mutational mechanism is not immediately attributed to a proposed extracted signature. The link to mechanism is now only in our Discussion.
2. In the discussion, we have added further explanation as to why we consider each mutational process primarily associated with our extracted signatures. We also add an explanation on why we think there are clearly further mutational processes at work (such as the mentioned polymerase error) and why we still are confident in our signature extractions despite this (see additions to pages 17 and 18).
3. While it is very difficult to absolutely attribute a mutational signature to its real process, there is a growing body of evidence to support each of these three mechanisms being important contributors to SARS-CoV-2's ongoing evolution. The repeatable extraction of these signature patterns with contexts and substitution types that are similar to those of the aforementioned mutational processes gives us confidence in these assignments. (Suppl. Figure S9, page 40)
4. Finally, to reflect the hypothetical nature of the signature's link to different host antiviral molecules, we have moderated the title to "*Is SARS-CoV-2's evolutionary capacity mostly driven by host antiviral molecules?*".

In Figures 4, 5 and 6, the authors assess the proportion of mutations associated with their extracted signatures. According to these plots and the author-assigned drivers of the signatures, we are led to believe that the viral polymerase has not made a single unrepaired replication error during the pandemic as all mutations are suggested to be driven by APOBEC, ADAR and ROS. While the proofreading exonuclease encoded by SARS-CoV-2 can recognise replication errors, it clearly won't repair all mistakes and polymerase errors will contribute to mutational spectra. This again highlights why assigning signatures to APOBEC, ADAR and ROS without further support is a major issue.

Response: We have added more discussion surrounding replication error and its effects on the extracted signatures. We acknowledge that the mutational signatures we see do not explain all of the observed mutations. Many changes in the dataset are not included in the signatures, and we cannot explain all of the mutations in the dataset, since the exposures do not equate to the full number of mutations at each epidemic week. Our expanded Figure 6 now quantifies the error for the unobserved mutation categories to show the mutations we are missing due to being not summarised by either of the 3 signatures. We are of the opinion this expansion now clearly shows the effect of the observed mutational processes while acknowledging that processes like polymerase error clearly occur but we do not have the power to detect them. (Pages 11-14)

There is a need for further validation of the patterns of mutational signatures through time. For example, the authors suggest that signature 2 causes the majority of mutations in Alpha in early

2021. If this were true, we'd expect to see a very high proportion of A-to-G and T-to-C mutations within this time period, as these are the dominant mutation types in signature 2. The authors include Figure A3 showing counts of unique substitutions per week split by mutation type (although this doesn't seem to be referenced in the text). There doesn't seem to be a large increase in the proportion of mutations that are A-to-G or T-to-C during early 2021, nor a decrease in C-to-T which would be expected if signature 1 is reducing within Alpha. While only a fraction of global sequences at this time are Alpha, I'd still expect to see some impact in this plot. To support the signature patterns, the authors should show similar plots to Figure A3 but broken down by variant and continent, to complement the signature plots in Figure 5c-d.

Response: We have created plots for each variant subset to show the substitution patterns as requested in Suppl. Figure S7. As the signature decreases suggest, we do see a reduction in C->T and a rather high quantity of A->G and T->C changes in the Alpha lineage that support the signature exposure plots. This ratio between C->T changes and A->G/T->C changes in Alpha is much greater than observed in other lineages, which would explain the increased exposure of Signature 2 relative to Signature 1 in Alpha. The same plots for continents can be found in the revised Suppl. Figure S8.

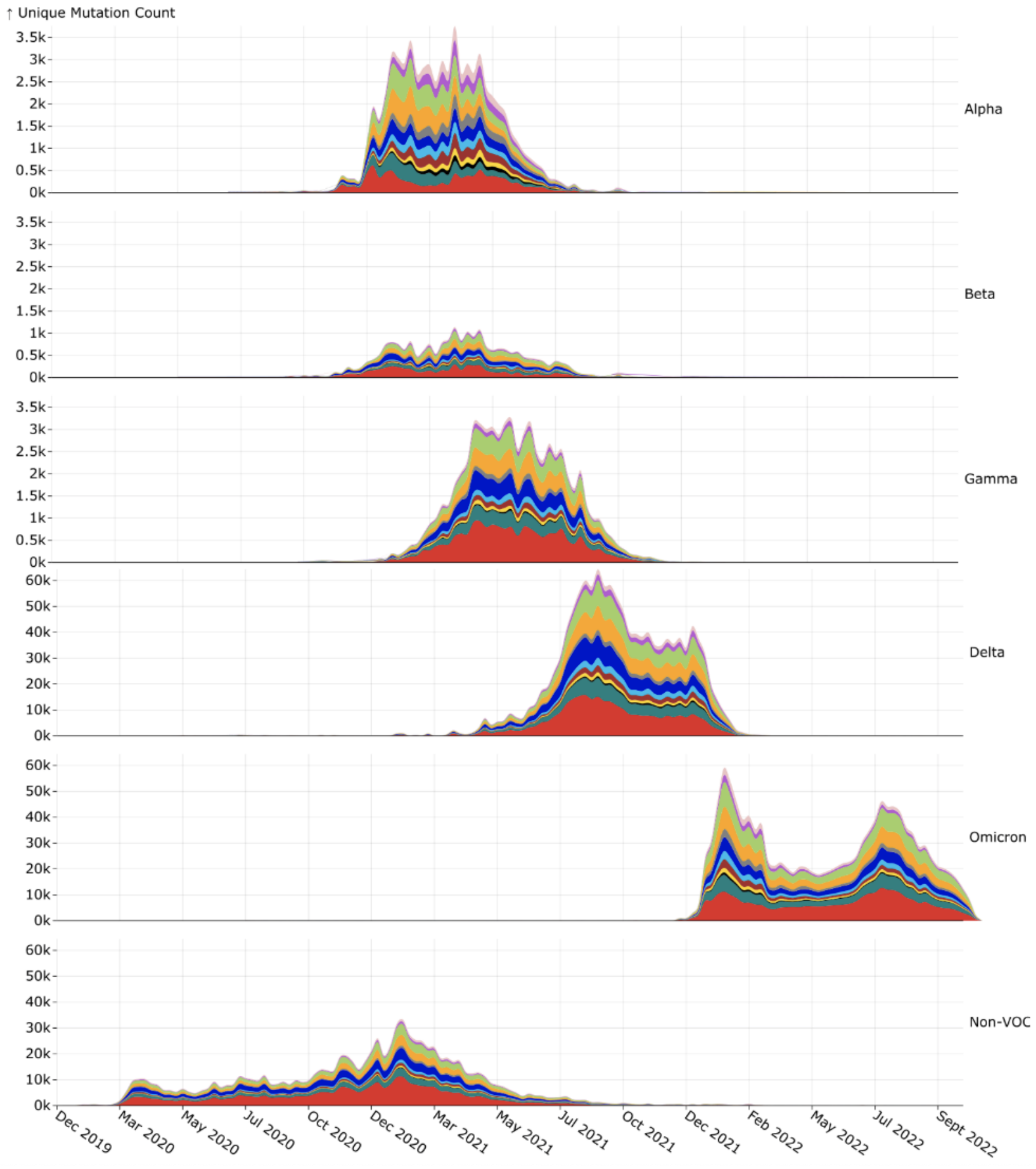

Section 2.5 examining the drivers of particular amino acid changes is highly problematic. Assigning mutations to signatures based on their mutation type is not possible. For example, C-to-T mutations are present in each of the three extracted signatures. The authors cannot therefore identify a C-to-T mutation and state that it was driven by signature 1, as there is a reasonable probability it could have been driven by signature 2 or 3, or by a currently unidentified signature. It's also unclear why the authors would include G-to-A mutations in

signature 1 here when G-to-A is a major contributor to signatures 2 and 3. The interpretations of this data are then highly problematic – for example, the authors state "Clearly, this APOBEC-like process was frequently active prior to the Alpha VOCs emergence"; this is based on the branch leading to Alpha having a proportion of C-to-T mutations. To assign these to signature 1 purely because they are C-to-T mutations is not possible. As outlined above, the drivers of these signatures are not supported, so this statement combines an unreliable assignment of mutations to signatures with an unreliable assignment of the driver of this signature and attempts to make a major conclusion about the processes contributing to variant generation. This is not reliable. Another example is "The high density of ADAR RBD mutations in a variant that has emerged as the ADAR signature has increased may suggest that the ADAR mutational process has driven the emergence of the Omicron variant." The authors are making major conclusions here that are not supported by reliable data. These are examples but section 2.5 is largely formed of the same analysis and conclusions. It may be possible to generate a statistical model that can take a set of contextual mutations and provide some estimate of the processes that have contributed to their generation based on mutation types and their surrounding contexts but failing this, section 2.5 does not provide reliable insights.

Response: Thanks for this comment. We agree that using simple substitution as a way to attribute mutations to their process is naive, so instead we have used a maximum likelihood approach to assign mutations to signatures in a more agnostic fashion. A mutation is now attributed to a signature rather than a specific process. The mutations are assigned using the maximum likelihood of a mutation and its context being assigned to one of the three signatures. Using the trinucleotide context C[C->T]G as an example, the likelihood function is P(C[C->T]G | Signature), which correspond to the probability bars in the extracted signatures. This ensures that the mutation allocation is agnostic to pre-existing expectations and keeps the analysis purely data-driven (see additions to page 11-13).

The methods section is light on detail, but my reading of the methods is that the authors calculated mutational spectra by comparing individual sequences from GISAID against an "ancestral lineage reference sequence". It is not clear from the methods whether this sequence is the ancestor of the same lineage the sequence is assigned to or the parental lineage as the authors use an example of sequences from lineage B.1 being compared against the reference for lineage B. Did the authors use one reference per lineage? Or were ancestral references only included for some lineages? Importantly, how were mutations counted? For example, if we take a cluster of related sequences within lineage B.1 that share 3 mutations that were acquired since the lineage B.1 ancestor, are these 3 shared mutations only counted once? Or are they counted in each of the sequences in the cluster? Each mutation should be counted on each phylogenetic branch on which the mutation occurred. Only counting each mutation once will undercount convergent mutations while counting each mutation in each sequence in which it occurs will result in highly inaccurate spectra. The methods here need to be clarified and, if necessary, updated.

Response: We have added clarity to the lineage reference section in particular, and have added a supplementary figure S3 showing examples of which sequences use which lineage

reference. We have also added a section clarifying the use of unique mutations to extract mutational signatures and discuss its impact on the counting of convergent mutations. We believe this now adequately explains any ambiguity that previously existed with this section. We use one reference per lineage and mutations are counted once within a lineage. We don't cluster sequences within lineages (this was included as a reference to how another paper attempted to solve the same problem). While undercounting convergent mutations in a lineage is possible, convergence will still be captured between lineages, and overcounting inherited mutations will cause, as mentioned, highly inaccurate signature extraction. (See page 23,24,25 and 39 for these changes.)

How were the surrounding contexts of each mutation identified? Were these identified from the same "ancestral lineage reference" or from the Wuhan-Hu-1 sequence?

Response: Mutational context was identified using the lineage reference so that acquired mutations would be included in the context as well, which would not be the case if the Wuhan-1 reference was used.

The authors don't appear to have rescaled the calculated spectra by triplet availability in the genome, which is a necessary step to obtain reliable mutational spectra. For example, if the genome contains 1000 CCT contexts but only 100 GCG contexts, a C-to-T mutation would be far more likely to occur in a CCT context than a GCG context. Therefore to obtain a mutational spectrum that represents the actual mutational processes, it is necessary to divide the mutation counts by the number of occurrences of the starting triplet, as has been carried out in previous publications on SARS-CoV-2 spectra (for example PMID 37185044).

Response: We have now generated the mutational signatures using normalised counts as requested. This now gives 2 sets of signatures, one set showing the patterns of mutations given the reference sequence genomic biases, and one set agnostic of triplet biases. Both are now discussed when referring to mutational process attribution since the normalised signatures give a better indication of process context specificity, while unnormalised show the real probability of change given the viral sequence. Both are of interest clearly, since one can help to attribute the real chance of a mutation editing a context, whereas the other can help identify signature aetiology (see additions to Page 17, 18,19, 25 and 36).

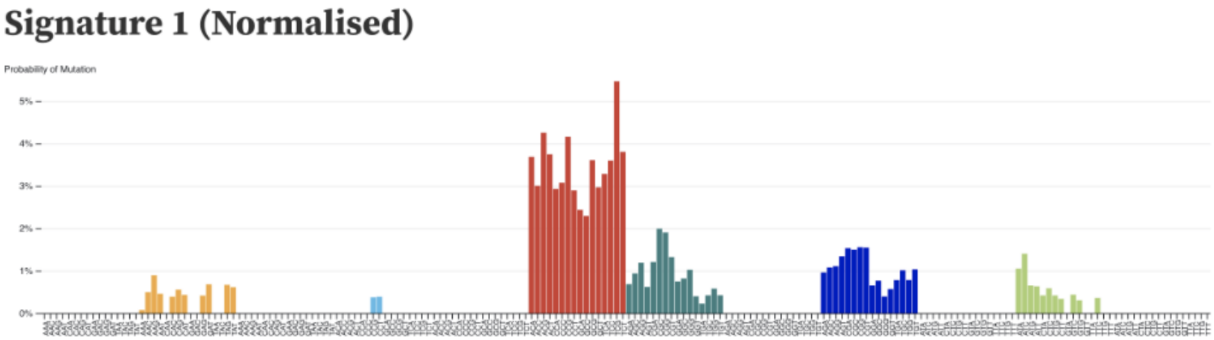

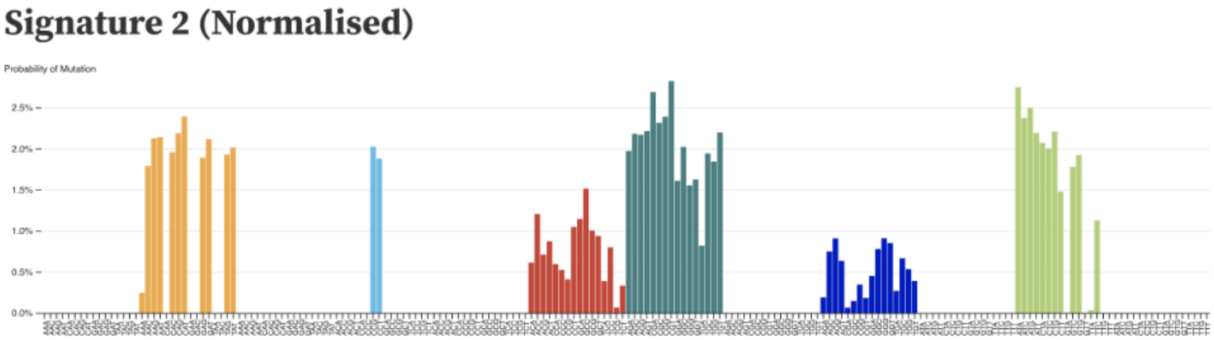

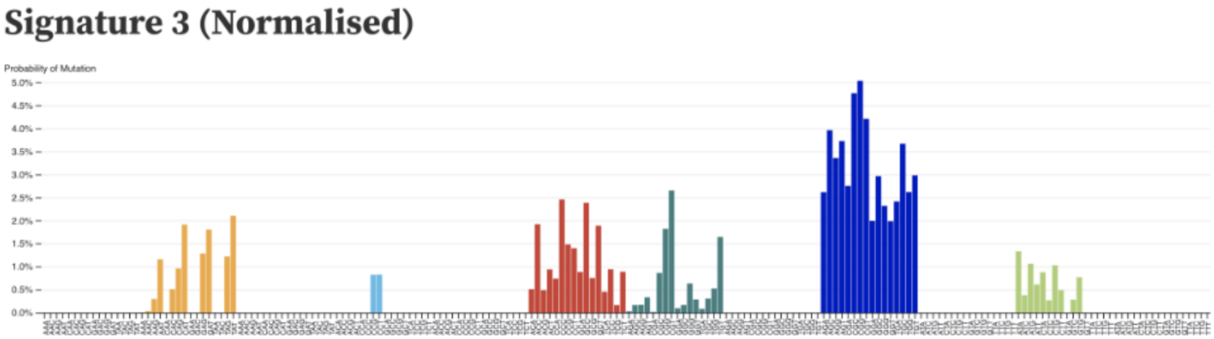

I'm not sure what the novel takeaways are from section 2.1 as all of the statements in this section (number of lineages, dominance of certain lineages, waves of different variants, infection rates, VOCs increasing infection rates) have been extensively covered in previous publications and public dashboards/resources.

Response: We acknowledge that the topics discussed have been previously covered in other literature and resources. The intention behind including section 2.1 was to provide a consolidated overview and context for readers. It was also aimed at setting the stage for the subsequent sections of our paper where we delve into more specific and potentially novel findings. This helps us provide a narrative for known driving factors as well as other non- variant factors

Section 2.2 should include sensitivity analyses. There are significant challenges associated with the analyses in section 2.2 as testing and case reporting differ hugely by country. This may be possible to account for at the level of individual countries where there is good knowledge of the

testing and reporting practices but at the continent level this appears to be a major challenge and it's not clear that the data is reliable enough to enable effective modelling. Additionally, the authors only examine four predictor variables and exclude other factors that are very likely to have an effect, for example previous infection burden. These factors may contribute to the inability of the models to completely fit the data in the insets in Figure 1, for example the fit in Oceania appears poor. A sensitivity analysis to assess the impact of poor estimates of cases in some regions and the impact of missing variables would be needed to trust the output from this modelling (or citations to previous papers that have done this if such papers are available). Furthermore, the authors state that "fitted mean response values with predictor values as the input resembled the rise and fall of infection cases". There is not currently a statistical assessment to support this and it is not clear that this is the case from examining the data in Figure 1; a statistical assessment of the model fits should be carried out to support this statement.

Response: Thank you for the thoughtful feedback on Section 2.2 and the concerns raised therein. We have addressed each point raised:
1. Challenges at the continent level: We recognize the disparities in testing and case reporting across countries. To address this, we have refined our continent-level analysis. Specifically, the influence of each country on the continent-level OxCGRT index is now weighted according to its proportionate impact on the continent's 14-day average daily case count. We believe that this is not exhaustive but better captures the unique dynamics at play within each continent and offers a better understanding.

2. Accounting for true burden: While we now employ the Human Development Index (HDI) to adjust reported cases (citing https://www.ncbi.nlm.nih.gov/pmc/articles/PMC9408439/), we acknowledge that this may not fully capture the true infection burden. Several factors underlie the actual burden, and while HDI provides one approach, it is by no means exhaustive. However, it serves as a useful proxy to bridge some of the data gaps.

3. Inclusion of additional predictor variables: We agree with this observation regarding the omission of potential influential factors such as previous infection burden. Predictor variables were included based on data completeness over the study period. We have revised our model to integrate previous infection burden which enhances their predictive power. We acknowledge these predictors aren't comprehensive and discuss this limitation

4. Model fit for Oceania: the predictor variables globally in our initial analysis did not appear to have a good fit in Oceania. Reviewing our model (adjusting continent level cases by HDI, including additional predictor variables and weighting the continent-level OxCGRT index by the proportionate impact on daily cases), greatly improved the overall fit.

5. Uncertainty in predictions: We include confidence errors in our predictions. We also now report R squared values for every continent and country included in the analysis, which gives an indicator of the goodness of fit.

Figure 2. Continent level.

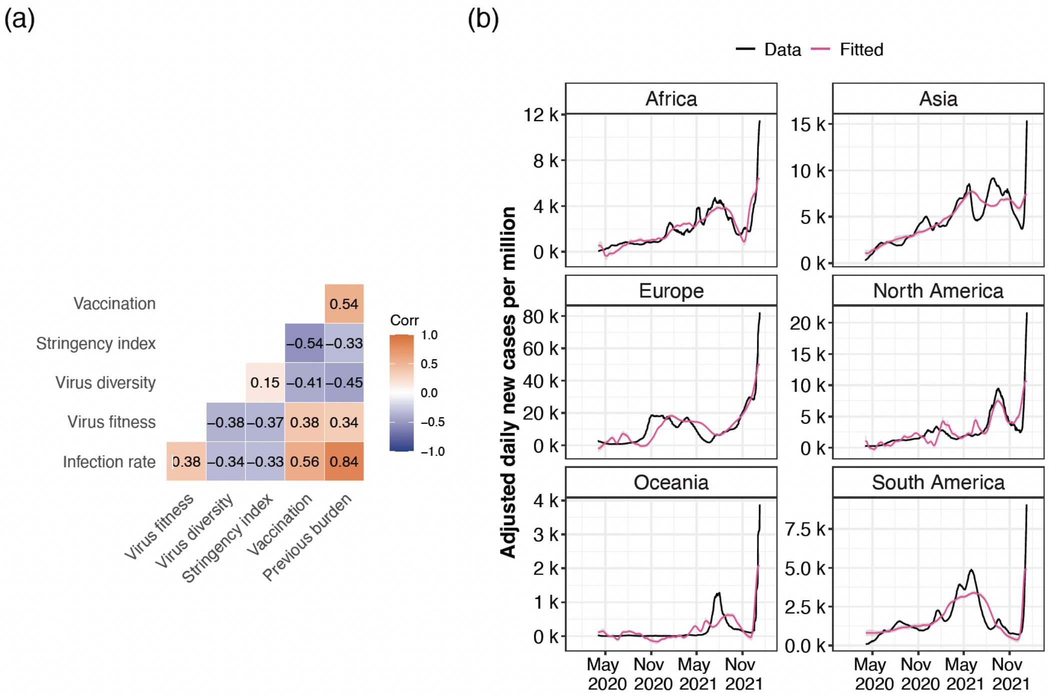
(a)
(b)
Vaccination
Stringency index
Virus diversity
Virus fitness
Infection rate
0.54
−0.54 −0.33
0.15 −0.41 −0.45
−0.38 −0.37 0.38 0.34
0.38 −0.34 −0.33 0.56 0.84
Corr
1.0
0.5
0.0
−0.5
−1.0
Virus fitness
Virus diversity
Stringency index
Vaccination
Previous burden
Data
Fitted
Adjusted daily new cases per million
Africa
Asia
Europe
North America
Oceania
South America
May 2020
Nov 2020
May 2021
Nov 2021

Suppl. Figure S2. country level regression model fitting.

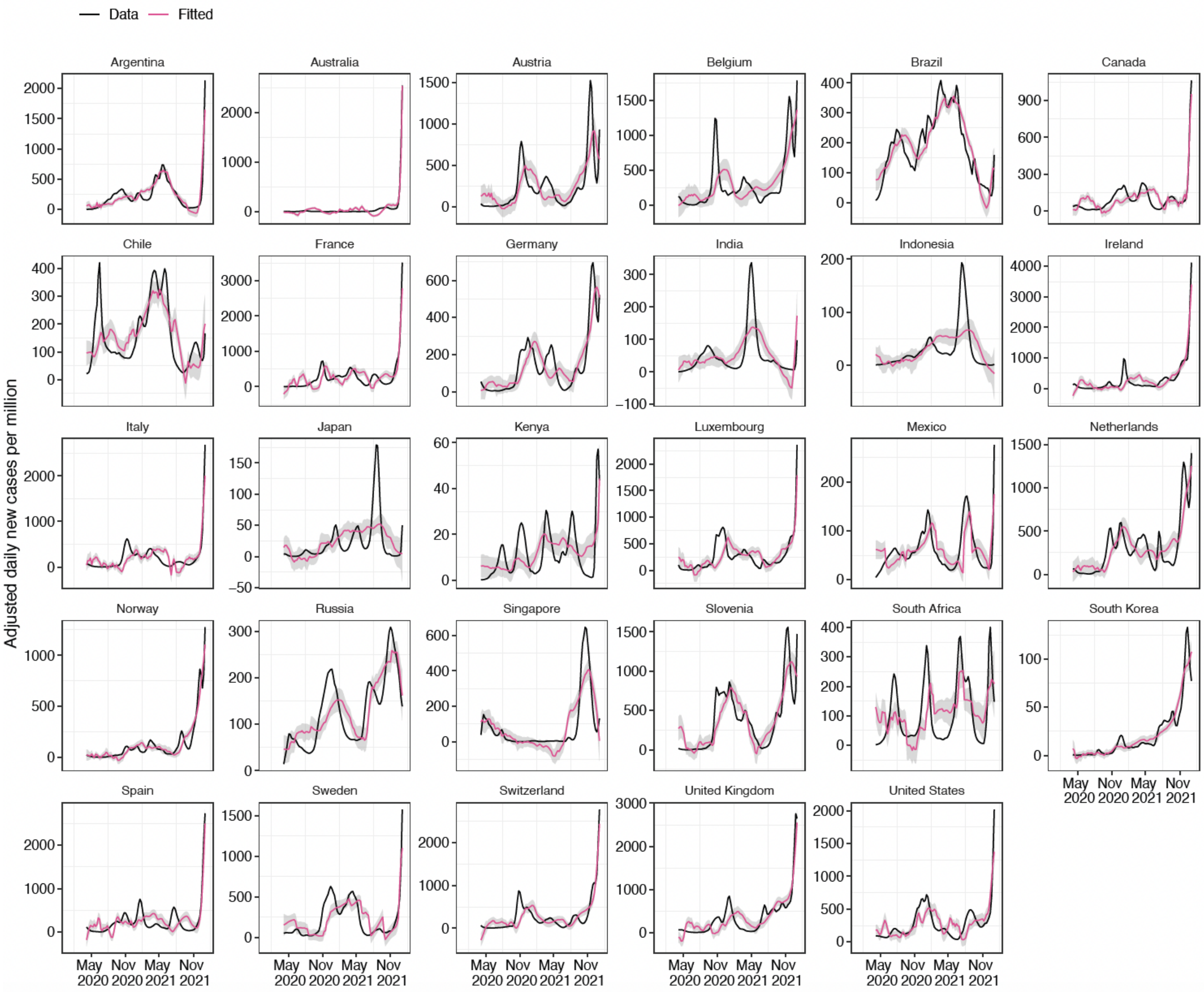

Suppl. Table A3. Effect of public health measures (government stringency and vaccination) and viral properties (diversity and fitness) on infection rates at continent level.

		**Africa**	**Asia**	**Europe**	**North America**	**Oceania**	**South America**
**Diversity score**	Estimate	832.4	1503.85	40828.15	15101.25	140.69	-339.15
	Pr	0.1	0.34	1.77E-26	4.89E-24	0.08	0.36
	Std.Error	511.12	1560.94	3663.33	1433.58	79.41	372.11
	t.value	1.63	0.96	11.15	10.53	1.77	-0.91

**Fitness score**	Estimate	730.01	1036.67	1680.87	506.22	228.52	1042.5
	Pr	3.60E-89	2.68E-23	2.84E-07	1.25E-08	1.42E-100	4.55E-86
	Std.Error	30.55	100.12	323.91	87.76	8.8	43.77
	t.value	23.89	10.35	5.19	5.77	25.98	23.82
**Government index**	Estimate	37.15	-14.76	28.13	-184.69	7.14	-4.28
	Pr	1.73E-12	0.11	0.41	3.10E-69	4.65E-09	0.43
	Std.Error	5.16	9.1	33.85	9.26	1.2	5.41
	t.value	7.21	-1.62	0.83	-19.95	5.95	-0.79
**Intercept**	Estimate	-2789.53	1931.43	-9258.87	12349.05	-573.13	1223.64
	Pr	6.31E-19	0.02	4.59E-06	3.99E-68	3.09E-09	0.01
	Std.Error	303.5	831.44	2003.05	625.45	95.26	445.29
	t.value	-9.19	2.32	-4.62	19.74	-6.02	2.75
**Previous burden**	Estimate	0.01	0.01	0.01	0	0.01	0
	Pr	2.05E-54	5.90E-28	4.32E-68	2.21E-05	3.79E-35	1.03E-12
	Std.Error	0	0	0	0	0	0
	t.value	17.24	11.51	19.75	-4.27	13.2	7.3
**Vaccine doses**	Estimate	-0.09	-0.01	0.04	0.04	-0.01	-0.02

	Pr	6.37E-22	2.32E-11	5.07E-23	8.25E-17	1.86E-06	3.06E-19
	Std.Error	0.01	0	0	0	0	0
	t.value	-10.01	-6.81	10.28	8.56	-4.82	-9.32
**Residual S.E**		750.68 on 600 DF	1253.55 on 626 DF	5772.52 on 637 DF	1445.60 on 640 DF	273.20 on 606 DF	713.85 on 536 DF
**R squared**		0.79	0.73	0.75	0.72	0.66	0.7
**Abbreviations:**<br>**DF = degrees of freedom**<br>**S.E = Standard error**							

Suppl. Table A4. Effect of public health measures (government stringency and vaccination) and viral properties (diversity and fitness) on infection rates at national levels

Country	Intercept	Government index	vaccine_doses	Fitness score	Diversity score	Previous burden	R squared
Argentina	-391.02	2.42	-0.01	318	431.67	0	0.86
Australia	-270.08	2.73	-0.06	111.8	46.23	0.11	0.96
Austria	-318.86	3.29	0.36	-84.88	450.04	0.01	0.54
Belgium	-57.36	-1.17	0.04	4.37	373.74	0.01	0.54
Brazil	-140.05	2.76	-0.01	38.45	25.43	0.01	0.81
Canada	-1011.36	11.85	0.02	175.49	509.21	0	0.8
Chile	550.61	-5.93	-0.03	65.26	-31.82	0.01	0.53
France	-2038.69	9.04	0.06	641.03	2842.56	0.01	0.75
Germany	165.55	-1.99	0.01	-85.54	-42.31	0.02	0.69
India	-28.35	-0.23	-0.03	76.94	116.53	0	0.44
Indonesia	36.03	0.21	-0.01	2.08	-70.45	0	0.35
Ireland	-749.06	0.74	0.01	388.13	1223.4	0.02	0.84
Italy	-1647.1	13.98	0.03	374.47	1419.41	0.01	0.72
Japan	23.36	0.48	0	-0.4	-75.31	0	0.28

Kenya	20.71	-0.14	-0.02	8.81	-4.36	-0.01	0.32
Luxembourg	-791.67	7.3	0.04	224.14	763.4	0.01	0.59
Mexico	-208.26	3.46	0	37.41	-35.13	0.01	0.46
Netherlands	-343.6	1.24	0.33	-45.1	535.95	0.01	0.66
Norway	-337.8	1.91	0.03	12.56	418.25	0.02	0.9
Russia	145.48	-0.76	0.02	-32.88	-72.81	0.01	0.67
Singapore	12.02	2.67	0.02	-97.97	-237.39	0.01	0.69
Slovenia	-471.64	5.5	0.09	-145.49	460.28	0.01	0.73
South Africa	-123.02	4.85	-0.05	39.76	-338.02	0	0.32
South Korea	9.48	0.14	0.01	6.17	-27.66	-0.01	0.89
Spain	-1292.19	2.61	0.07	476.79	2058.39	0.02	0.7
Sweden	-1084.41	18.58	0.47	80.66	155.09	0	0.61
Switzerland	-651.84	0.61	0.05	226.8	1498.23	0.02	0.77
United Kingdom	-417.36	-3.26	0.03	263.37	1717.29	0.01	0.79
United States	-1427.87	16.63	0.06	145.45	1108.11	0	0.62

Minor comments

Results section 2.1, paragraph 1, line 5 – table A2 shows some Pango lineages and some WHO variant names, the "13 Pango lineages" should be updated to reflect this.

Response: This has been addressed

Figure 1 – from the legend, the bars show biweekly proportions of common lineages. Does the whitespace show the proportion of sequences from other lineages? Please specify.

Response: Yes we have updated our text to state this more clearly.

Figure 1 – not all insets can be read, it would be useful to update the figure to make these clearer.

Response: We have updated our figures, moving the insets into Figure 2b for better legibility

Methods section 4.1 – what were the cutoffs for “excessive mutations/deletions”? Please describe.

Response: The cutoff for filtering out hypermutated sequences was 175 mutations in coding regions or more than 69 different deletions, the cutoffs were manually determined after evaluation of the mutation/deletion distribution and selecting the point where sequence counts were consistently observed in single digits, this resulted in 1,852 sequences being filtered out.

Methods section 4.2, paragraph 1, lines 2-3 – “We collected continuous dependent vari- ables that changed with every calendar date did not remain constant for finite amounts of time.” It’s not clear what this sentence means, is it possible to update?

Response: We've revised the sentence to better convey our intention. The updated sentence now reads: “We collected continuous dependent variables reported on a daily basis.” We believe this modification offers a straightforward understanding of our data collection method.

Methods section 4.2, paragraph 1, lines 7-8 – missing vaccination data was handled as no vaccines were administered. Are these data points at times where it is reasonable to believe that no vaccines were administered (i.e. before vaccine rollouts in the respective countries)? This would be useful to state.

Response: In response to this query, the instances where vaccination data was missing primarily corresponded to periods before vaccine rollouts in the respective countries. Thus, it was reasonable to treat these missing data points as periods where no vaccines were administered. We have updated the text to reflect this.

Methods section 4.2 – some more details on the estimation of virus fitness would be useful. How were the mutations in each lineage defined? How is the sum of fit mutations performed - is each “fit” mutation observed counted as 1 regardless of frequency in the population?

Response: Mutations were defined against the Wuhan-Hu-1 sequence. Each fit mutation in a sequence was counted as 1 (frequency depended on the number of sequences with that mutation), and this was normalized to the number of sequences generated in a given location. We have now updated our text to give more detail.

Methods section 4.7 – what was the NMF run on? Was this run on the set of spectra from all epidemic weeks? Please expand this section to make clear. A supplementary figure showing the reasoning for three signatures being chosen as optimum would be useful.

Response: The NMF was run on 100 bootstrapped epidemic week datasets for each set of signatures, i.e., 2-10. The set of individual samples was bootstrapped and then converted into the epidemic week pseudo-sample datasets which ensured that each set of pseudosamples were created from a different set of sequences drawn from the original data. We have added some text to clarify this further and as requested have created a graphical figure showing the methods process down to the evaluation of the signature N selection in Suppl. Figure S4.

Figure 2 – while it's clear which panel is which, panels A and B aren't labelled in the figure currently.

Response: Thank you for pointing this out. This has now been addressed

Figure 3 – not all mutation types are represented in the signatures (for example A-to-C to T-to-G). These mutation types do occur within SARS-CoV-2 mutations (for example see PMIDs 37185044 and 37039557). This should be stated and included in the discussion around the activities of signatures through time, as it is likely that not all mutations are driven by the extracted signatures.

Response: This is an excellent point, we have added a number of sections to address this and stress that while the signatures cover much of the observed mutations, other mutational processes could still be in play.

The use of mutation nomenclature is not always consistent – for example C->T or CT. Please update.

Response: This has now been updated.
