# Supplementary material for: Mutational signature dynamics indicate SARS-CoV-2’s evolutionary capacity is driven by host antiviral molecules
Source: PLoS Comput Biol. 2024 Jan 25;20(1):e1011795. doi: 10.1371/journal.pcbi.1011795 (PMC10868779; doi:10.1371/journal.pcbi.1011795)
Supplement: Attachment — Submitted filename: Lamb-etal-response-to-reviewers-Dec2023.pdf [file pcbi.1011795.s016.pdf]

Response to reviewers, PCOMPBIOL-D-23-01052R1

Reviewer 1

Comments

- Please move the statement on lines 122-123 “Note, SARS-CoV-2 has an RNA genome… signature notations” to immediately after line 119 where thymine is first mentioned.
Response: The sentence has now been moved.

- I advocate for swapping the placement of figure 3 and supplementary figure 5 in the manuscript. The normalized values are a better representation of the mutational signature and do not detract from the overall takeaway of the impact of these substitutions on the three signatures. Doing so also strengthens the authors’ claims regarding the context-specific induction of the APOBEC-like signature. I recognize that my feelings regarding composition normalization are more stringent than that of the rest of the field and, for that reason, I treat this as a suggestion and not a requirement for the authors. Doing the replacement would require some editing of the discussion, where normalization results are generally presented after the raw values (which is sensible if the normalization is in the supplementary material; e.g., lines 363-364, 380-381, etc.)
Response: Thanks for the comment, we have switched the figures and re-arranged the discussion to match this change.

- “Alphas” (sic) is missing a possessive apostrophe on line 420.
Response: The apostrophe has been added.

Reviewer 2

Comments

Discussion lines 384-386 – exposure of human DNA to ROS has shown quite strong context dependence, for example see figure 3B in PMID 30982602
Response: The ROS context dependence is now mentioned in the manuscript, along with some brief discussion.

Figure 5B – a legend showing the colours of the 3 signatures would be useful for clarity
Response: The figure legend has now been added.

Figure S7 – a legend showing the colour corresponding to each mutation type would be useful, as is currently included in Figure S8
Response: The figure legend has now been added.
